# Restorative effect of endurance exercise on behavioral deficits in the chronic mouse model of Parkinson's disease with severe neurodegeneration

**DOI:** 10.1186/1471-2202-10-6

**Published:** 2009-01-20

**Authors:** Konstantinos Pothakos, Max J Kurz, Yuen-Sum Lau

**Affiliations:** 1 Department of Pharmacological and Pharmaceutical Sciences, University of Houston, Houston, Texas 77204, USA; 2 Department of Health and Human Performance, University of Houston, Houston, Texas 77204, USA

## Abstract

**Background:**

Animal models of Parkinson's disease have been widely used for investigating the mechanisms of neurodegenerative process and for discovering alternative strategies for treating the disease. Following 10 injections with 1-methyl-4-phenyl-1,2,3,6-tetrahydropyridine (MPTP, 25 mg/kg) and probenecid (250 mg/kg) over 5 weeks in mice, we have established and characterized a chronic mouse model of Parkinson's disease (MPD), which displays severe long-term neurological and pathological defects resembling that of the human Parkinson's disease in the advanced stage. The behavioral manifestations in this chronic mouse model of Parkinson's syndrome remain uninvestigated. The health benefit of exercise in aging and in neurodegenerative disorders including the Parkinson's disease has been implicated; however, clinical and laboratory studies in this area are limited. In this research with the chronic MPD, we first conducted a series of behavioral tests and then investigated the impact of endurance exercise on the identified Parkinsonian behavioral deficits.

**Results:**

We report here that the severe chronic MPD mice showed significant deficits in their gait pattern consistency and in learning the cued version of the Morris water maze. Their performances on the challenging beam and walking grid were considerably attenuated suggesting the lack of balance and motor coordination. Furthermore, their spontaneous and amphetamine-stimulated locomotor activities in the open field were significantly suppressed. The behavioral deficits in the chronic MPD lasted for at least 8 weeks after MPTP/probenecid treatment. When the chronic MPD mice were exercise-trained on a motorized treadmill 1 week before, 5 weeks during, and 8–12 weeks after MPTP/probenecid treatment, the behavioral deficits in gait pattern, spontaneous ambulatory movement, and balance performance were reversed; whereas neuronal loss and impairment in cognitive skill, motor coordination, and amphetamine-stimulated locomotor activity were not altered when compared to the sedentary chronic MPD animals.

**Conclusion:**

This study indicates that in spite of the drastic loss of dopaminergic neurons and depletion of dopamine in the severe chronic MPD, endurance exercise training effectively reverses the Parkinson's like behavioral deficits related to regular movement, balance and gait performance.

## Background

Parkinson's disease (PD) is a neurodegenerative disorder afflicting millions of people especially in the growing aging population. Patients with PD are typically debilitated with symptoms of muscular rigidity, impaired movement, loss of balance, and tremor at rest. There is no medical cure for PD. Current therapies are mainly targeted at masking or reducing disease symptoms; however, such measures are only temporary. A desirable approach for intervening or slowing down the course of neurodegeneration in PD would be the search for alternative strategies that either protect against neuronal loss (neuroprotection) or rejuvenate the defective cells (neurorestoration). Exercise and balance training in the early disease stage have been reported in a number of clinical studies showing overall improvement in muscle strength, balance, daily activities, motor performance, and ambulation in PD patients [1-3]. However, the effect of exercise on neurological and behavioral manifestations has not been consistently presented in human PD or in laboratory models of PD. Therefore, more research is necessary for validating the long-term benefits of endurance exercise in PD.

Since its discovery in the early 1980's, 1-methyl-4-phenyl-1,2,3,6-tetrahydropyridine (MPTP) has been extensively used by researchers to produce animal models of PD [4]. While humans and non-human primates are most susceptible to MPTP in developing PD-like symptoms [5,6], mouse models are widely used for studying the neurological and pathological mechanisms underlying the degeneration of nigrostriatal dopaminergic neurons. Our laboratory has developed and characterized a chronic mouse model of Parkinson's disease (MPD) in which mice are treated with MPTP and probenecid over a 5 week period [7,8]. Probenecid inhibits the urinary and neuronal clearance of MPTP and its metabolites, which causes a potentiated loss of neurons in the substantia nigra and persistently impairs dopamine (DA) transmission for at least 6 months [7,8]. In contrast to the most commonly used acute and subacute MPTP mouse models of PD in which neurological and behavioral deficits are short-lived and spontaneously reversed soon after the treatment, the chronic MPD has long-lasting neurological deficits showing many features resembling that of the Parkinson's syndrome, which include significant loss of cells, DA content and terminal DA uptake in the nigrostriatal neurons, detection of early neuronal apoptosis and delayed α-synuclein-positive inclusion bodies in the substantia nigra pars compacta [9,10].

An important dimension of different MPTP mouse models is the manifestation of behavioral deficits that resemble Parkinsonian signs and symptoms making them useful for testing potential therapeutic agents. Given the multitude of models varying in the amount of MPTP administered and dosing schedules, published reports have delineated an inconsistent picture of animal behaviors relevant to Parkinsonism, especially when mice are observed in the open field. While a majority of studies have shown reduced locomotor and rearing activities in the MPTP-treated mice, other studies have reported either no change in behavior or a development of hyperactivity [11]. The discrepancies existed in behavioral results could also be contributed by the differences in the duration of observation or the time points after MPTP treatment at which the assessment is made. Diminished cognitive abilities such as slowed responses and dementia in human PD have not been replicated in MPTP rodent models. There is a paucity of studies exploring potential cognitive deficits in MPTP-treated mice using tasks that relate to nigrostriatal function. However, following MPTP or 1-methyl-4-phenyl-pyridinium treatment, behavioral tasks such as passive and active avoidance, T-maze have been utilized to detect memory/recognition skills that are associated with the function of hippocampus, frontal cortex or olfactory bulb [12-14].

In the present study, we first examined the motor and cognitive skills in the chronic MPTP/probenecid-treated MPD by monitoring their gait pattern, their performance on the challenging beam and grid apparatus, in the cued and spatial versions of Morris water maze (MWM), as well as in the open field. We then exercise-trained the chronic MPD and compared the outcome of endurance exercise on their behavioral performances with that of the sedentary chronic MPD.

## Results

### Exercise effect on neurological deficits in the severe chronic MPD

When mice were treated with 10 injections of a near maximal regimen of MPTP hydrochloride (25 mg/kg) and probenecid (250 mg/kg) over 5 weeks, there was a persistent loss of substantia nigra tyrosine hydroxylase immunopositive cells and depletion of striatal DA at least for a survival span of 3–24 weeks [7,8]. Relevant to the present study, which focused on animal behavioral monitoring and analyses, for instance at 12 weeks after chronic MPTP/probenecid treatment, the striatal DA level in the chronic MPD was 1.92 ± 0.12 ng/mg tissue (N = 6), which was significantly depleted when compared with the level found in the chronic probenecid-treated controls, 14.85 ± 0.62 ng/mg tissue (N = 5), *P *< 0.0001. Continuous exercise training for 12 weeks after MPTP/probenecid treatment did not significantly raise the level of striatal DA (2.67 ± 0.29 ng/mg tissue, N = 6). Likewise, immunohistochemical and stereological analyses revealed a 72% loss of the tyrosine hydroxylase positive neurons in the substantia nigra pars compacta of the severe chronic MPD and exercise training did not significantly reverse the cell loss.

### Exercise effect on gait pattern consistency

**Figure 1:**
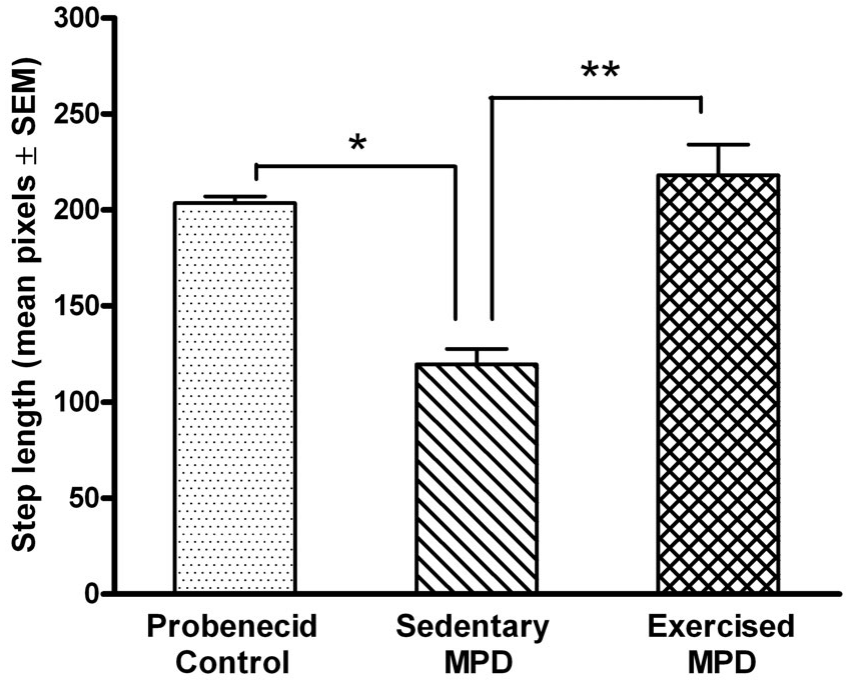
**Effect of exercise on the step length in the chronic MPD**. The mean step length in the sedentary chronic MPD mice 12 weeks after MPTP/probenecid treatment was significantly shorter than the probenecid-treated controls (*P = 0.0001). Continuous exercise for 12 weeks after MPTP/probenecid treatment in the chronic MPD completely reversed the step length deficit as detected in the sedentary chronic MPD group (**P = 0.0001). The mean step length in the control group and exercised chronic MPD group were not statistically different.

The animal step length and gait pattern consistency in the control (N = 8), sedentary (N = 9), and exercise-trained MPD (N = 5) were measured and compared. As shown in Fig. 1, the statistical omnibus F indicated that there was a significant difference in the averaged step length between the three groups of animals [F (2, 19) = 255.78, *P *= 0.0001]. The post-hoc analyses revealed that the sedentary chronic MPD had a significantly shorter step length than the chronic probenecid-treated control group (*P *= 0.0001). Continuous exercise training for 12 weeks after MPTP/probenecid treatment in the chronic MPD group effectively reversed the step length deficit; thus, the averaged step length exhibited in the exercise-trained chronic MPD was significantly longer than that of the sedentary chronic MPD group (*P *= 0.0001) and was not different from that of the control group of animals.

**Figure 2:**
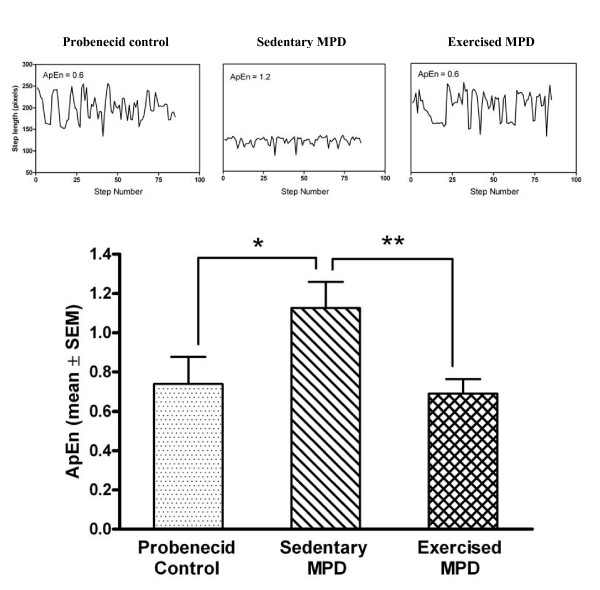
**Effect of exercise on the gait pattern in the chronic MPD**. The representative pattern of step length time series from a probenecid control mouse, a sedentary chronic MPD mouse, and an exercised chronic MPD mouse 12 weeks after MPTP/probenecid treatment were shown at top. The calculated gait pattern certainties in terms of approximate entropy (ApEn) for the three groups of animals were respectively shown in the bar graph below. A larger ApEn value denotes a more variable movement pattern, which was significantly demonstrated by the sedentary chronic MPD when compared with the control group of animals (*P = 0.0001). The ApEn value for the exercised chronic MPD was significantly lower suggesting a more consistent gait pattern than that of the sedentary chronic MPD (**P = 0.007). The gait pattern consistency (ApEn value) in the control group and exercised chronic MPD group were not statistically different.

The chronic MPD animals demonstrated a noticeable loss of gait pattern consistency (Fig. 2). The statistical omnibus F showed that there was a significant difference in the gait pattern consistency over the entire period of recording among the three groups of animals [F (2, 19) = 13.39, *P *= 0.0001]. The post-hoc analyses indicated that the sedentary chronic MPD mice had a more inconsistent gait pattern (represented by a higher value of ApEn) than that of the probenecid-treated control animals (*P *= 0.0001). When the chronic MPD group was exercise-trained for 12 weeks after MPTP/probenecid treatment, their consistency of stepping pattern returned to normal; thus, the gait pattern as expressed in mean ApEn for the exercised chronic MPD was significantly lower than that of the sedentary chronic MPD (*P *= 0.007) and was not statistically different from that of the control group of animals. These data suggest that the chronic MPD mice characteristically display a shorter step length and an inconsistent gait pattern associated with severe dopaminergic neurodegeneration. Long-term exercise training restored the gait performance in the chronic MPD.

### Exercise effect on the cued version of Morris water maze

**Figure 3:**
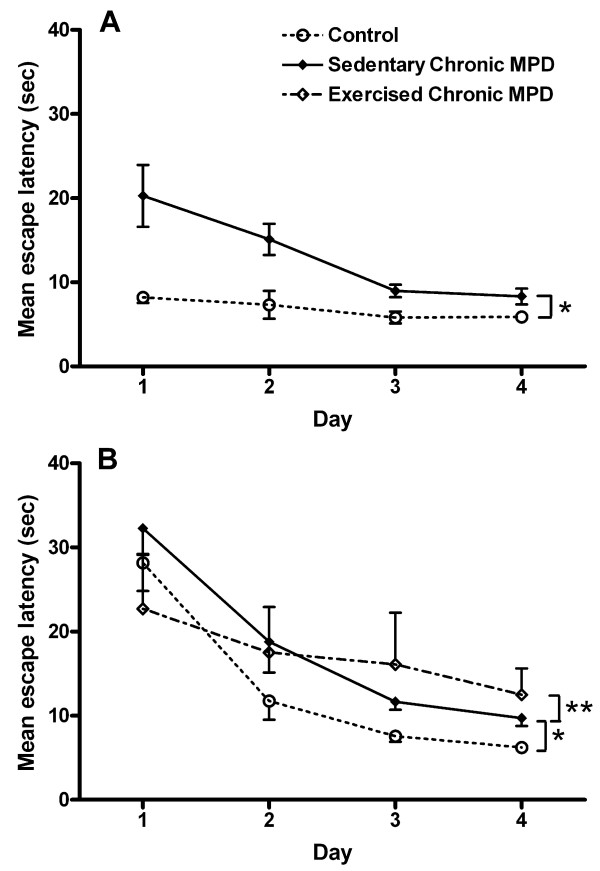
**Exercise effect on learning of the cued version of the Morris water maze (MWM) in the chronic MPD**. (A) One day and (B) 10 weeks after MPTP/probenecid treatment, the chronic MPD group of mice showed significant impairment in learning the cued version of the MWM when compared with the controls (A, *P = 0.01 and B, *P = 0.012, respectively). (B) Continuous exercise for 10 weeks after MPTP/probenecid treatment did not alter the learning deficit for the cued version of the MWM in the chronic MPD (**P = 0.557, when compared to the sedentary chronic MPD).

One day after the last injection of MPTP/probenecid, the chronic MPD (N = 10) demonstrated a significant impairment in acquiring the cued version of the water maze task showing significantly longer latencies when compared with that of the control group of animals (N = 4), [F (1, 12) = 8.96, *P *= 0.01] (Fig. 3A). Similar results were found when the test was given to the chronic MPD group 10 weeks after the chronic MPTP/probenecid treatment (N = 9); thus, they showed a significant deficit in learning the task when compared to the control group (N = 9), [F (1, 16) = 8.26, *P *= 0.012] (Fig. 3B). Continuous exercise for 10 weeks after MPTP/probenecid treatment in the chronic MPD (N = 5) did not significantly improve the animal's performance in the cued version of the MWM when compared with the sedentary chronic MPD mice (N = 9) [F (1, 12) = 0.37, *P *= 0.557] (Fig. 3B). These findings suggest that the chronic MPD exhibits an immediate as well as a long-term deficit in habitual learning that is linked to the severe nigrostriatal neurodegeneration. Long-term exercise training does not reverse the habitual learning deficit in the chronic MPD.

### Exercise effect on the spatial reference version of Morris water maze

**Figure 4:**
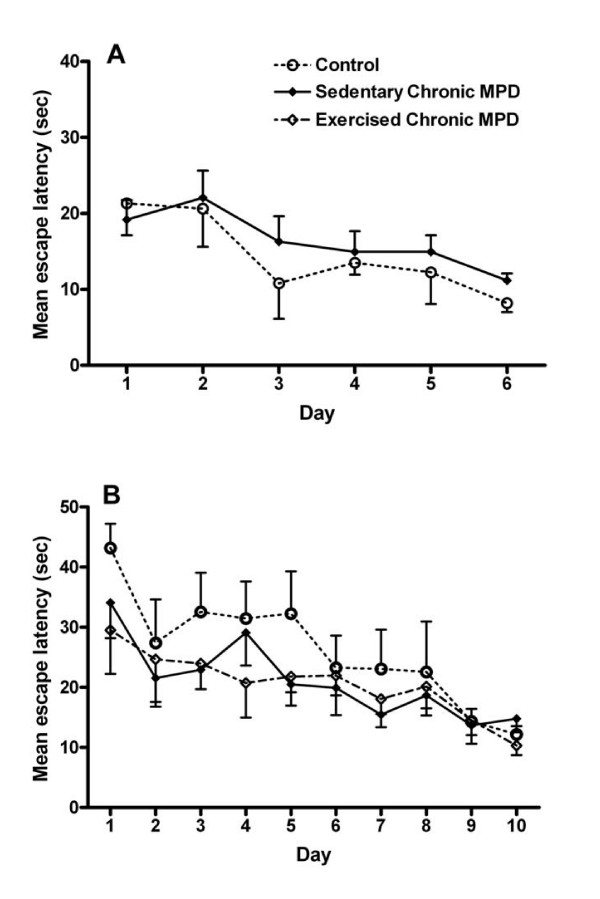
**Exercise effect on learning of the spatial reference version of the MWM in the chronic MPD**. (A) One week and (B) 8 weeks after MPTP/probenecid treatment, the chronic MPD group of mice showed no impairment in learning the spatial reference version of the MWM when compared to the controls. (B) Continuous exercise for 8 weeks after MPTP/probenecid treatment did not alter the learning for the spatial reference version of the MWM in the chronic MPD.

In contrast to the cued version of water maze task, no significant difference in the spatial reference version of water maze learning was detected between the control and the chronic MPD either short-term (1 week, N = 9, *P *> 0.05) (Fig. 4A) or long-term after the MPTP/probenecid treatment (8 weeks, N = 8, *P *> 0.05) (Fig. 4B). Exercise training for 8 weeks after MPTP/probenecid treatment in the chronic MPD did not significantly modify this behavior (N = 8 for sedentary chronic MPD; N = 5 for exercised chronic MPD) (Fig. 4B). These results indicate that chronic MPTP/probenecid treatment does not affect the hippocampus in a manner that would impair the animal's ability to acquire the spatial reference learning task. Thus, the baseline hippocampal function which has been shown to be critical for accomplishing this task [15] appears to be intact in the chronic MPD and is not affected by exercise training.

In order to exclude the possibility that chronic MPTP/probenecid treatment might influence the overall ability of swimming in mice, we further compared the swimming speed in the two groups of animals. Briefly, a narrow (6 cm) path was created with two wooden planks along the diameter (120 cm) of the water maze without an escape platform. The time latency for each mouse to swim across the entire path based on two trials (ITI = 30 sec) was averaged and statistically analyzed. The mean time for the control group (N = 9) and chronic MPD group (N = 10) to swim across the path were 4.89 ± 0.21 s and 4.80 ± 0.16 s, respectively, which were not statistically different. This observation confirms that MPTP/probenecid treatment in the chronic MPD does not significantly impair the animal's physical ability for swimming that might interfere with their ability to locate the escape platform in the water maze.

### Exercise effect on challenging beam and grid tasks

**Figure 5:**
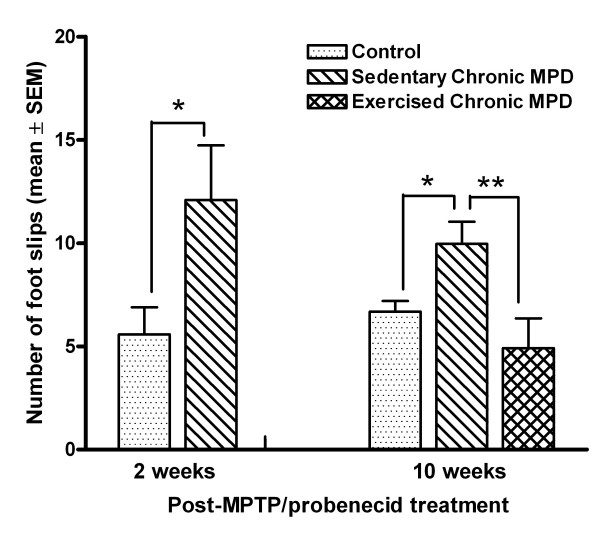
**Exercise effect on balance performance on a challenging beam in the chronic MPD**. Two and 10 weeks after MPTP/probenecid treatment, the chronic MPD group of mice had significantly more limb slips than the controls while traversing the challenging beam (*P = 0.041 and *P = 0.013, respectively). The chronic MPD, when continuously exercised for 10 weeks after MPTP/probenecid treatment, had significantly less foot slips on the challenging beam than the sedentary chronic MPD (**P = 0.01).

We used the challenging beam and grid tasks in this study to examine the animal's balance and motor coordination skills. Two weeks after MPTP/probenecid treatment, the chronic MPD group (N = 10) displayed a significantly higher number of foot slips on the challenging beam than that of the control group (N = 10), [F (1, 18) = 4.86, *P *= 0.041] (Fig. 5). Same results were obtained from the chronic MPD 10 weeks after treatment (N = 10), which had significantly more foot slips on the challenging beam than the probenecid-treated control group (N = 10) [F (1, 18) = 7.52, *P *= 0.013] (Fig. 5). Continuing exercise training for 10 weeks after MPTP/probenecid treatment (N = 7), these animals had significantly less foot slips on the challenging beam when compared with the sedentary chronic MPD (N = 10) [F (1, 15) = 8.18, *P *= 0.01] (Fig. 5)

**Figure 6:**
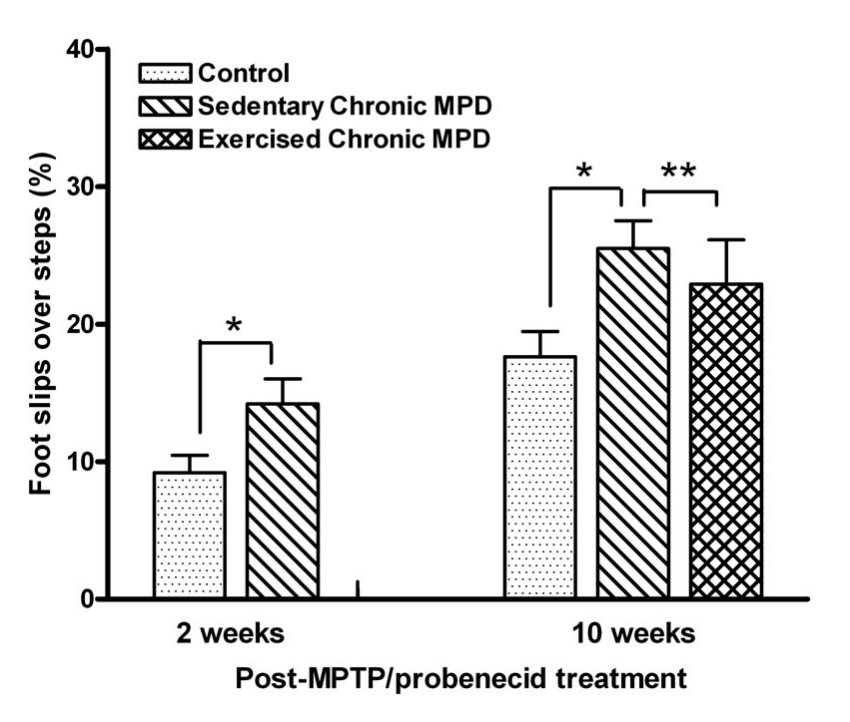
**Exercise effect on motor coordination on a walking grid in the chronic MPD**. Two and 10 weeks after MPTP/probenecid treatment, the chronic MPD group of mice had significantly higher percentage of limb slips over steps than the controls while walking on a perforated grid (*P = 0.037 and *P = 0.01, respectively). Continuous exercise for 10 weeks after MPTP/probenecid treatment did not significantly improve the grid walk performance in the chronic MPD (**P = 0.485).

Using a grid walk test, we also observed that the chronic MPD (N = 10) had a significantly higher percentage of foot slips through the grid openings than that of the control mice 2 weeks (N = 10) [F (1, 18) = 5.05, *P *= 0.037], and 10 weeks (N = 10) [F (1, 18) = 8.27, *P *= 0.01] after chronic MPTP/probenecid treatment (Fig. 6). However, continuous exercise training for 10 weeks after MPTP/probenecid treatment did not significantly improve their grid walk performance (N = 10 for sedentary chronic MPD; N = 7 for exercised chronic MPD) (Fig. 6).

The data from the challenging beam and grid tasks together demonstrate that the chronic MPD mice have impaired balance and motor coordination skills, which persisted for at least 10 weeks after MPTP/probenecid treatment. Exercise training improves the balancing skill but fails to restore the motor coordination skill in the chronic MPD.

### Exercise effect on open field behaviors

**Figure 7:**
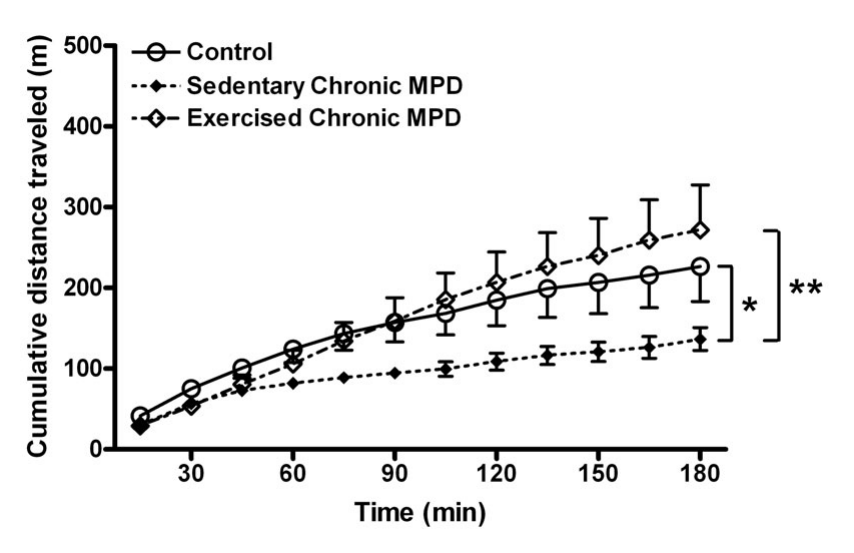
**Exercise effect on the spontaneous locomotor activity in the chronic MPD**. The horizontal movement expressed as cumulative distance traveled by each animal was recorded over 180 min. The spontaneous locomotor activity in the chronic MPD mice 11 weeks after MPTP/probenecid treatment was significantly lower than that of the chronic probenecid-treated control mice (*P = 0.03). Continuous exercise for 11 weeks after MPTP/probenecid treatment in the chronic MPD completely reversed the spontaneous movement deficit as detected in the sedentary chronic MPD group (**P = 0.02).

We further recorded the locomotor behaviors in mice in the open field 11 weeks after chronic probenecid or MPTP/probenecid treatment. To obtain the basal levels of activity, control and chronic MPD were injected with saline and their ambulation was observed for 3 hours. The total distance traveled (m) by the chronic MPD under basal conditions (N = 10) was significantly lower than that of the chronic probenecid-treated control animals (N = 9), [F (1, 17) = 5.58, *P *= 0.03) (Fig. 7). When the chronic MPD animals were exercise trained for 11 weeks after MPTP/probenecid treatment (N = 7), they completely recovered from the spontaneous movement deficit when compared to the sedentary chronic MPD (N = 10), [F (1, 15) = 6.84, *P *= 0.02] (Fig. 7).

**Figure 8:**
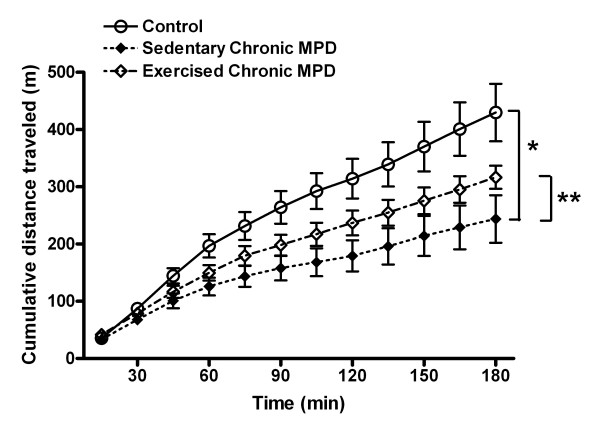
**Exercise effect on the amphetamine-induced locomotor activity in the chronic MPD**. The amphetamine (3 mg/kg, i.p.)-induced locomotor activity in the chronic MPD mice 11 weeks after MPTP/probenecid treatment was significantly lower than that of the chronic probenecid-treated control mice (*P = 0.009). Following 11 weeks of exercise training, no statistically significant recovery of the amphetamine-stimulated movement was observed when comparing to the sedentary chronic MPD (**P = 0.186).

Three days after monitoring the spontaneous movement, each animal received a single dose of amphetamine (3 mg/kg, i.p) and the drug-induced locomotor activities were recorded for 3 hours. The amphetamine-challenged ambulatory activity detected in the chronic MPD (N = 10) was also significantly lower than that of the probenecid-treated control mice (N = 9), [F (1, 17) = 8.67, *P *= 0.009] (Fig. 8). Continuous exercise training for 11 weeks after MPTP/probenecid treatment (N = 7) did not significantly change the amphetamine-stimulated movement when compared to the sedentary chronic MPD (N = 10), [F (1, 15) = 1.92, *P *= 0.186] (Fig. 8). Our data that showed diminished spontaneous and amphetamine-stimulated behavioral responses in the chronic MPD would implicate a long-term locomotor dysfunction corresponding to the neurological dopaminergic deficit in the nigrostriatal tract. Exercise training effectively reverses the spontaneous movement deficit but does not significantly reverse the depression of amphetamine-stimulated movement as exhibited in the chronic MPD.

## Discussion

### Behavioral manifestations in the chronic MPD

The present study examined and compared the cognitive and motor skills in the chronic MPD with severe nigrostriatal neurodegeneration shortly after and beyond 8 weeks of Parkinson's syndrome development. In association with marked loss of nigrostriatal dopaminergic cells and DA content, we observed that the chronic MPD showed significant deficits in (1) gait consistency pattern; (2) the learning capacity when challenged with the cued version of water maze; (3) balance and motor coordination skills on the challenging beam and walking grid; (4) the spontaneous and amphetamine-stimulated locomotor behaviors in the open field. Noticeably, these diminished behavioral responses lasted for at least 8–12 weeks after MPTP/probenecid treatment. The inhibited behavioral manifestations were apparently associated with the severe loss of nigrostriatal transmission, since the function of the hippocampus remained unaffected as shown by their unaltered ability to learn the spatial reference version of the water maze.

One of the clinical features of advanced PD is truncal rigidity that creates difficulty for affected patients to maintain an upright posture and a normal gait; thus, while walking they tend to take small steps in a hasty pace, which would result in frequent falls [16]. Totally replicating the PD-liked postural and gait deficits in quadrapedal rodents is not expected to be physiologically feasible. At best, a gait analysis technique that measures and compares the variability of stride length and stepping pattern may be applied to rodent models of PD [17]. Indeed, as demonstrated in the present study, the gait analysis revealed that the severe chronic MPD has a relatively shorter stepping length and a less consistent gait pattern certainty than the non-Parkinsonian mice. Therefore, this technique does provide a reliable approach for assessing the experimental outcomes and for predicting the severity of neurological lesions comparable to the clinical gait variability as reported in human PD [18,19].

Human studies through several neuropsychological tests have reported that PD patients without dementia have a reduced ability for acquiring cue-based probability learning tasks with no impairment in the process of recollective memory; these findings suggest that neostriatum plays an important role not only for learning and performing motor skills but is also essential for processing the gradual, incremental learning of associations known as habitual learning [20,21]. In the rodent literature, the cued version of the MWM is a stimulus-response or habitual task, which requires an intact network of the basal ganglia in order for successfully acquiring the task [45]. Therefore, lesions present in the basal ganglia would suppress the learning ability of rodents to associate a unique cue located on the platform as a visual sign with a safe landing place in the water maze. In the present study and to our knowledge for the first time, we tested and confirmed that a deficit in the cued version of the MWM could be demonstrated not only shortly after MPTP/probenecid treatment but could also be associated with long-term and severe nigrostriatal neurodegeneration in the chronic MPD [9]. Considering that various attempts for behaviorally characterizing different MPTP mouse models of PD have produced inconsistent results so far, the findings of this study indicate that the cued version of the MWM would be a useful test for assessing short-term and long-term habitual learning deficit associated with MPTP-induced lesions in the basal ganglia of mice.

As mentioned earlier, the spatial reference version of the MWM requires the rodents to use various cues from the environment in the close vicinity of the maze for locating the escape platform and this skill is dependent on the integral function of the hippocampus. Several dissociation studies have clearly distinguished the two cognitive learning systems independently involving the hippocampus and the basal ganglia [22,23]. When MPTP was administered centrally or through nasal inhalation, rats developed short-term impairment in their learning of the cued version but not the spatial reference version of the MWM task [24-26]. This notion is substantiated by the observations of the current study, in which we detected no change in the performance of the spatial reference version of the MWM in mice 1 week or 8 weeks after MPTP/probenecid treatment. Hence, there is no evidence suggesting that chronic MPD mice have developed learning deficits linked to the hippocampus, and we have not found published reports describing a direct toxic effect of MPTP on the hippocampus that would dysregulate the cognitive function.

In this study, we strove to determine whether the order of administering the two different versions of MWM to the same animal would impact the outcome of learning the respective task. We therefore randomly altered the sequence for conducting the two versions of MWM in the chronic MPD. The results were consistent regardless which order of the task that we used first, because it is apparent that they are functionally regulated by two distinctive neuronal network systems. Furthermore, we performed a control experiment illustrating that the chronic MPD mice did not have physical impairment that would hinder their ability for swimming, since the amount of time for them to swim across the maze without visual cues was indifferent from that of the control mice.

The extrapyramidal system of the basal ganglia has long been recognized for its control and refinement of motor performance and coordination [27,28]. We examined the short-term and long-term motor and balancing skills in the chronic MPD animals by monitoring their walk on a challenging beam and a wired grid. The challenging beam task has been successfully used for demonstrating motor deficit in a transgenic mouse model of PD that overexpresses α-synuclein [48]. The grid walk task has been widely used for testing animal models for spinal injury, ischemia and traumatic brain injury [29,49]. Both tests confirmed that the chronic MPD mice exhibited motor and balance deficits not only within 2 weeks but also persisted for at least 10 weeks after the MPTP/probenecid treatment when compared to the control animals. The prolonged motor dysfunction displayed by the chronic MPD mice appears to resemble the movement disability and imbalance in the human PD.

The gradual loss of nigrostriatal neurons and depletion of the neurotransmitter, DA have been considered as the basis of locomotor deficit in Parkinson's disorder, which can be demonstrated in laboratory animals by monitoring their ambulatory movement in the open-field. In the present study, we measured both the spontaneous and the amphetamine-stimulated movement in the chronic MPD and we found that these animals exhibited a long-term deficit in horizontal movement whether the striatal DA system was unstimulated or stimulated. These observations support the concept that the level of DA in the striatal terminal vesicles is considerably depleted as demonstrated in the chronic MPD [7,8]; thus the normal release and stimulated release of the stored vesicular DA through the indirect action of amphetamine are both diminished. Recently, similar abnormalities in amphetamine-challenged horizontal movement in the open field that correlate with increasing levels of striatal DA depletion in an acute MPTP mouse model of PD have also been reported implicating that the amphetamine-stimulated assessment of movement in PD models can be validated [30].

Following an acute single injection with a moderate dose of amphetamine, it is known that the drug is taken up by the terminal dopaminergic transporter before it triggers the release of stored DA from vesicles. Nevertheless, the amphetamine effect is not specific to the dopaminergic neurons. It can be taken up by other monoamine terminal transporters in the same fashion and the involvement of norepinephrine and serotonin release may further modify the ambulation of the animal [31]. It is also possible that amphetamine exerts its effects on locomotion via the terminal DA_2 _autoreceptors [32]. MPTP is shown to reduce the DA_2 _receptor mRNA expression [33] and mice with knockout DA_2 _receptors have exhibited reduced spontaneous locomotion [34,35]. Taken together, the reduced spontaneous and amphetamine-stimulated ambulatory movement detected in the chronic MPD could be contributed by either or both the depletion of DA content in the storage vesicles and a down-regulation of the DA_2 _receptors. The latter possibility involving the DA_2 _receptors requires further investigation in the chronic MPD mice.

### Effect of endurance exercise on behavioral deficits in the chronic MPD

Following the investigation of behavioral deficits in the chronic MPD, we further studied the effect of endurance exercise on their behavioral deficits. Although continuous exercise for 12 weeks after MPTP/probenecid treatment did not significantly reverse the neurological deficit in the nigrostriatal dopaminergic system in this severe animal model of Parkinsonism, we are gratified to detect that endurance exercise training significantly invigorated the spontaneous movement and restored the gait pattern consistency and balance in the chronic MPD. However, exercise training did not have the same positive impact on the motor coordination and cognitive learning deficit that are also associated with the severe nigrostriatal neuron degeneration.

Behavioral assessment of exercise effect has been reported in several animal models of Parkinsonism. In the rat model of Parkinsonism induced by 6-hydroxydopamine, either voluntary running or treadmill paced exercise attenuated DA loss in the striatum with or without significant recovery of behavioral deficits [36-38]. The neuronal recovery in 6-hydroxydopamine-treated rats triggered by exercise is associated with an increase of the striatal glial cell line-derived neurotrophic factor [39]. In an acute mouse model of Parkinsonism induced by MPTP, treadmill exercise ameliorates behavioral deficits and reverses several striatal dopaminergic indices including the loss of DA, TH-immunoreactivity, and DA transporter levels when compared to the sedentary Parkinsonian animals [36]. In another study, high-intensity treadmill exercise in acute MPTP-treated mice leads to behavioral recovery; however, the striatal expression of DA transporter is down-regulated and the expression of TH is not changed [33]. Differences in the exercise results obtained from animal models of Parkinsonism could be due to experimental variables such as the age and species of the animal, the method and severity of the induced nigrostriatal lesion, and the type and intensity of the exercise regimen.

The chronic MPD that is induced by MPTP/probenecid and used in this study affords a neurodegenerative model for PD. With 10 doses of MPTP (25 mg/kg) plus probenecid (250 mg/kg), the chronic MPD attains a severe level of neurodegeneration showing neurochemical, histological, behavioral and pathological characteristics resembling that of PD at advanced stages [9]. Offering exercise training to the severe chronic MPD animals does not appear to produce neuroprotective or neuroregenerative effect to the dopaminergic neurons. It is not expected that exercise or other pharmacotherapeutic approaches would deliver such promises in the severe chronic MPD or advanced PD, in which the majority of neurons and neurotransmitters are already irreparably lost. However, it is so encouraging to learn based on the observations of this study that exercise training can effectively reverse certain behavioral deficits, like impaired movement, imbalance and inconsistent gait pattern that are associated with the Parkinsonian syndrome in spite of the existing severe loss of dopaminergic neurons and neurotransmitter. It is not clear why other behavioral abnormalities, like the impaired cognitive learning and motor coordination skills, are not recovered by exercise training. It is possible that intact nigrostriatal system and adequate level of striatal DA are obligatory for maintaining these behaviors, like in the case of open field study with amphetamine that its pharmacological action is dependent on the undamaged DA tract for eliciting movement hyperactivity. Additional studies are necessary for exploring and substantiating such a hypothesis.

## Conclusion

The results of this study demonstrate that the severe chronic MPD mice exhibit a wide range of behavioral phenotypes that represent symptoms comparable to that of the advanced PD. It is noteworthy that these behaviors persist and allow for several months of study. Combined with the neurochemical and pathological characteristics that we have previously reported in this chronic MPD, these behavioral tests would afford confirmation of dopaminergic neurodegeneration without requiring additional animal sacrifice for neurochemical and neuroanatomical analyses. The long-term neurological and behavioral deficits that are characterized in this chronic MPD would make this model more useful for exploring novel therapeutic targets for treating PD.

Endurance exercise has been widely advocated for maintaining healthy life in general, and for slowing down aging and neurodegenerative processes. The benefit of exercise on cardiovascular adaptation has been well studied and recognized; however, the information about exercise effect on neurological improvement and associated behaviors is still limited. This study demonstrates that in the severe chronic MPD with behavioral deficits comparable to the advanced Parkinsonian syndrome, exercise training can bring anomalous movement, balance and gait pattern back to the state of normalcy, even though the neurological indicators remain severely impaired. Therefore, exercise has a beneficial impact on reversing certain behavioral deficits associated with Parkinsonian-like neurodegeneration.

## Methods

### Chronic mouse model of Parkinson's disease

Four to six-month old, male, C57BL/6 mice (Harlan Sprague Dawley, Inc., Indianapolis, IN, USA) were housed in single cages with food pellets and water available *ad libitum*. The room was maintained at a constant temperature and humidity on a 12-h/12-h light/dark cycle. All experiments took place during light time. All animal treatments were carried out strictly in accordance to the National Institute of Health Guide for the Care and Use of Laboratory Animals (NIH Publications No. 80-23, revised 1996) and were approved by the Institutional Animal Care and Use Committees from the University of Houston. Our experimental procedures did not cause significant animal suffering. A total of 58 mice were used in the present study. For the behavioral testing, each animal within a cohort was tested only once for all the different tasks.

To prepare the chronic MPD with severe neurodegeneration, mice were injected with a total of 10 doses of MPTP hydrochloride (25 mg/kg/injection in saline, s.c.) in combination with an adjuvant, probenecid (250 mg/kg/injection dissolved in dimethyl sulfoxide, i.p.) as previously described [8]. The 10 doses were administered on a five-week schedule with an interval of 3.5 days between injections. Control mice were treated with probenecid only. MPTP hydrochloride and probenecid were purchased from Sigma Chemical Co. (St. Louis, MO, USA). Safety precautions for the use of MPTP during chemical preparation and animal injections were taken according to the procedures previously described [40].

### Endurance exercise training

A six-lane motorized rodent treadmill (Columbus Instruments, Columbus, OH, USA) was utilized for exercise training. Mice were exercise trained one week before, 5 weeks during the chronic MPTP/probenecid treatment, and exercise was continued after the last treatment till two day before experiment as shown in the following scheme:



The exercised group of animals was trained on the treadmill running for 5 days/week, 40 min/day at a speed up to 15 m/min (5 min at 6 m/min, 5 min at 9 m/min, 20 min at 12 m/min, 5 min at 15 m/min, and 5 min at 12 m/min) with 0° of inclination. Using this treadmill exercise protocol in our laboratory, the chronic MPD mice were able to go through the training with minimal requirement for external stimuli or manual prodding, yet the animals developed physical endurance after 4 weeks showing cardiorespiratory and metabolic adaptations comparable to those seen in human subjects undergoing continuous exercise training [41]. Sedentary mice did not exercise; however, they were transported daily to the training room so that they were exposed to the same environment as the exercised group of animals.

### Gait pattern measurement

The method for gait pattern determination and data expression in mice has been previously described [42]. Briefly, twelve weeks after chronic MPTP/probenecid treatment, mice were videotaped in the sagittal plane with a 60 Hz camera as they ran on a motorized treadmill at a speed of 10 m/min, 0° inclination. All gait data were collected on the same day and treadmill. The positions of the base of the tail, right fore and hind feet were manually digitized from the videos with commercially available motion capture software (ViconPeak, Centennial, CO). To account for any variation in the mouse's position on the treadmill, we calculated the displacement of the feet relative to the base of the tail. These newly defined feet positions were used to determine the selected step lengths for 85 consecutive steps. We calculated the mean step length used by each of the respective experimental groups.

Approximate entropy (ApEn), which is a regularity statistic that evaluates the likelihood that similar patterns in the time series will be present at a later time period was used for determining the consistency of the mouse's step lengths [43]. Accordingly, a time series with a more consistent pattern has an ApEn value closer to zero, while a time series with a less consistent or irregular pattern has an ApEn value closer to two. A higher ApEn has previously been validated and associated with movement dysfunctions found in the elderly and in individuals with PD [44].

### Cued and spatial versions of Morris water maze

The apparatus used was a circular white tub with an inside diameter of 120 cm. It was filled with water at room temperature (24°C), which was made opaque in color by dissolving a white non-toxic dye (Chromatemp, Chroma, Lititz, PA, USA). The water level reached at 15 cm below the top edge of the tub. The escape target was a white hexagonal platform with equilateral sides (7.5 cm per side), which was submerged 1 cm below the water surface. A day before testing was conducted, mice were pre-trained by exposing them to the water maze apparatus with the platform located in the center of it. Specifically, mice were first placed on the platform for 20 sec, followed by three consecutive trials consisting of releasing them in the water at a location approximately 10 cm away from the platform and allowing them to find the platform and stay on it for another 20 sec. If the mouse failed to locate the platform and/or to stay on it, then it was guided to complete the task by the experimenter.

We tested the animals in two different versions of MWM. The cued version of the MWM had an escape platform with a rod (12.5 cm in length, 1 cm in diameter) glued onto the platform and with a plastic ball (2.5 cm diameter) on the top of the rod. Both the rod and the ball had black and white stripes making them easily visible in the water maze environment. The position of the platform was altered for each trial. The cued version of MWM is a habitual learning task associated with the functionality of the striatal network [45]. This test was administered in our study one day (short-term) and 10 weeks (long-term) after the chronic MPD treatment.

The second version of the MWM that we used was the spatial reference test, in which the animal subjects relied on prominent spatial cues in the surrounding environment outside the maze, such as posters and laboratory objects on a desk in fixed positions. The escape platform had no visual cues and was kept in one position during all trials. The spatial reference version of the MWM measures the ability of cognitive learning that is associated with the integrity of the hippocampal network [46,47]. This test was administered in our study one week (short-term) and 8 weeks (long-term) after the chronic MPD treatment.

In both versions of MWM test, the mouse was released facing the wall of the water maze from a different starting point in each trial and was given 60 sec to find the platform. If the animal failed to find the platform, it would be gently led to it by the experimenter. In either case the animal was left on the platform for 20 sec, dried, and returned to its home cage. Each mouse was tested with 4 trials per day for 4 or 6–10 consecutive days (cued and spatial reference version, respectively) with an inter-trial interval (ITI) of 30 to 40 min. For statistical analysis, the latency (in seconds) for each animal to reach the platform based on four daily trials was averaged and considered as a single score for the day.

### Challenging beam and grid tasks

We further monitored the ability of the chronic MPD in maintaining balance and motor coordination on a challenging beam and a wired grid apparatus 2 weeks (short-term) and again 10 weeks (long-term) after chronic MPD treatment. The challenging beam was a 1 m long wooden beam suspended 23 cm above a bench top, which was covered with soft pads to protect the mouse in case of a fall. The beam was divided in four gradually narrowing sections (25 cm/section) leading to the mouse's home cage. The beam widths of the four sections were 3.5, 2.5, 1.5, and 0.5 cm in decreasing order [48]. The beam was covered with surgical tape that provided sufficient surface traction for the animals to walk on. There were 1 cm wide ledges hanging 1 cm below each side of the beam to encourage the mice to use their normal gait strategies even when their limbs slipped. All mice were pre-trained for two consecutive days (5 trials/day, ITI = 10–12 sec) on traversing the beam. On the third day, each mouse was given 5 trials (ITI = 10–12 sec) and the average number of videotaped limb slips per trial was used for statistical analysis. Slips were counted only while the mouse was in forward motion.

For the grid task, mice were allowed to explore and walk on a square metallic wired grid (30 cm × 30 cm) with square openings (1.25 cm/side) across the surface, which was mounted 50 cm above a bench top with soft pads underneath to protect the mouse in case of a fall. Each mouse was videotaped for 3 minutes while walking on the grid [49]. Only full limb slips and not partial foot entries through the grid openings were counted. The percentage of slips over total number of steps taken was used for analysis.

### Open field

We measured saline-injected baseline and amphetamine (3 mg/kg, i.p., Sigma, St. Louis, MO, USA)-challenged ambulatory activity in the open-field with an automated behavioral monitor equipped with an X-Y arrangement of infrared photoreceptor beams (16 per side) (Opto-Varimex, Columbus Instruments, Columbus, OH, USA). Each mouse was placed in a clear, open-top, square Plexiglas box (40 × 40 × 20 cm) within the monitor in a subdued room. As introduced earlier, since open field behaviors in mice shortly after MPTP treatment tend to be overly variable, in this study we monitored the open field ambulation limited to long-term (11 weeks) after the induction of chronic Parkinsonism. The total distance (m) traveled by each mouse was cumulated after every 15 min and plotted for a period of 3 hr.

### Statistical analysis

For gait pattern measures, two one-way ANOVA with Tukey HSD post-hocs were used to find statistical differences in the ApEn values and mean step lengths. The data collected for the water maze and open field behaviors were analyzed by using a mixed ANOVA with different time points as the within subjects factor and the treated and untreated groups as the between subjects factor. For the grid and challenging beam tasks, simple ANOVA was used (SPSS 12). All results were considered statistically significant when *P *< 0.05.

## Abbreviations

ApEn: approximate entropy; DA: dopamine; ITI: inter-trial interval; MPTP: 1-methyl-4-phenyl-1,2,3,6-tetrahydropyridine; MWM: Morris water maze; MPD: mouse model of Parkinson's disease; PD: Parkinson's disease.

## Authors' contributions

KP performed chronic MPD treatment, treadmill exercise, behavioral assessments, data analyses and co-wrote the manuscript. MJK contributed to the gait pattern analysis in normal, sedentary and exercised chronic MPD. YSL, the Principal Investigator of this project, participated in the development and supervision of the overall research design and protocols and co-wrote the manuscript.
